# A Pyrene Maleimide with a Flexible Linker for Sampling of Longer Inter-Thiol Distances by Excimer Formation

**DOI:** 10.1371/journal.pone.0026691

**Published:** 2011-10-20

**Authors:** Satomi Niwayama, Abdullatif S. Kassar, Tian Zhao, Roger B. Sutton, Guillermo A. Altenberg

**Affiliations:** 1 Department of Chemistry and Biochemistry, Texas Tech University, Lubbock, Texas, United States of America; 2 Department of Cell Physiology and Molecular Biophysics, and Center for Membrane Protein Research, Texas Tech Health Sciences Center, Lubbock, Texas, United States of America; University of Helsinki, Finland

## Abstract

Pyrene-containing compounds are commonly used in a number of fluorescence-based applications because they can form excited-state dimers (excimers) by stacking interaction between excited-state and ground-state monomers. Their usefulness arises from the facts that excimer formation requires close proximity between the pyrenes and that the excimer emission spectrum is very different from that of the monomers. One of many applications is to assess proximity between specific sites of macromolecules labeled with pyrenes. This has been done using pyrene maleimide, a reagent that reacts with reduced thiols of cysteines, but its use for structural studies of proteins has been rather limited. This is because the introduction of two cysteines at sufficiently close distance from each other to obtain excimer fluorescence upon labeling with pyrene maleimide requires detailed knowledge of the protein structure or extensive site-directed mutagenesis trials. We synthesized and tested a new compound with a 4-carbon methylene linker placed between the maleimide and the pyrene (pyrene-4-maleimide), with the aim of increasing the sampling distance for excimer formation and making the use of excimer fluorescence simpler and more widespread. We tested the new compound on thiol-modified oligonucleotides and showed that it can detect proximity between thiols beyond the reach of pyrene maleimide. Based on its spectroscopic and chemical properties, we suggest that pyrene-4-maleimide is an excellent probe to assess proximities between cysteines in proteins and thiols in other macromolecules, as well as to follow conformational changes.

## Introduction

Pyrenes can form an excited-state dimer (excimer) by stacking interaction between the excited monomer and ground-state monomer [1]. Compared with the monomer emission, the excimer emission is significantly red-shifted, which facilitates its detection [1]. During the last few years, this property has been used in the development of nanosensors based on nucleic acids (molecular beacons, aptamer sensors) [2]–[6]. Other applications include studies of lipid membranes, membrane fusion and protein structural studies [7]–[14]. One obvious application of the excimer formation is to assess proximity between specific sites of macromolecules labeled with pyrenes as well as to determine conformational changes that will affect pyrene stacking due to movements in macromolecules parts (changes in distances, rotations). With this idea in mind, cysteine residues in proteins were used as targets for the first time in 1973, using *N*-(1-pyrene) maleimide (pyrene maleimide) [15]. The use of pyrene maleimides for covalent linkage to proteins is particularly advantageous because the olefinic double bond of the maleimide reduces the fluorophore quantum yield, and therefore the reaction with the thiols can be followed by the increase in fluorescence [15], and removal of the unreacted reagent is not necessary. In spite of these advantages, the number of studies using pyrene maleimide for protein structural studies has been rather limited [8]–[13], [16]–[21]. One reason is that stacking of the pyrenes has a very limited distance range (usually the pyrenes have to be within 3 to 5 Å of each other for excimer formation). Therefore, introduction of two cysteines at sufficiently close distance from each other to obtain excimer fluorescence upon labeling with pyrene maleimide requires detailed knowledge of the structure or extensive site-directed mutagenesis trials. Tracking protein-protein associations using excimer fluorescence is not very practical for the same reason (*i.e.*, thiols in interacting proteins have to be very close to each other).

Although the discussion above centered on the distance between labeling sites, flexibility of the pyrene probe is as important as distance because excimer fluorescence occurs only with the correct stacking orientation. In addition, if the stacked pyrenes cannot reorient on excitation, emission will be quenched. Therefore, the absence of excimer emission in macromolecules labeled with pyrene maleimide can be a consequence of long inter-thiol distance and/or its relatively rigid structure. Here, we synthesized and tested a new compound with a 4-carbon methylene linker between the imide nitrogen of the maleimide and the pyrene (*N*-pyrenylbutyl maleimide), which we refer to as pyrene-4-maleimide. Our aim was to increase the sampling distance and efficiency of excimer formation, making the use of excimer fluorescence simpler and more widespread.

## Results and Discussion

**Figure 1:**
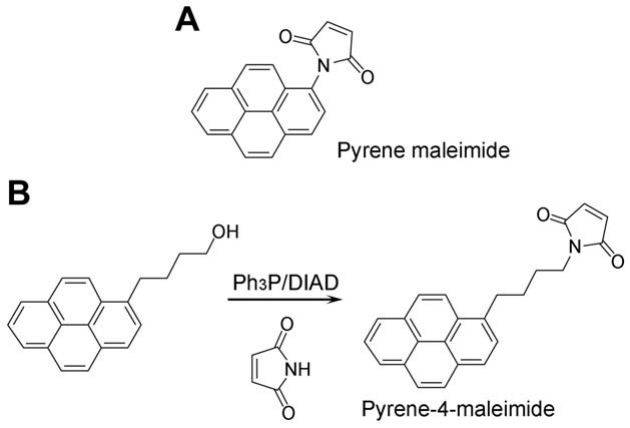
Schematic representation of the pyrene-4-maleimide synthesis and structure. A. Pyrene maleimide structure. B. Pyrene-4-maleimide synthesis route and structure. Ph_3_P: triphenylphosphine; DIAD: diisopropyl azodicarboxylate.

Pyrene-4-maleimide was synthesized from commercially available 1-pyrenebutanol and maleimide by the modified Mitsunobu reaction reported by Walker [22] (Fig. 1).

**Figure 2:**
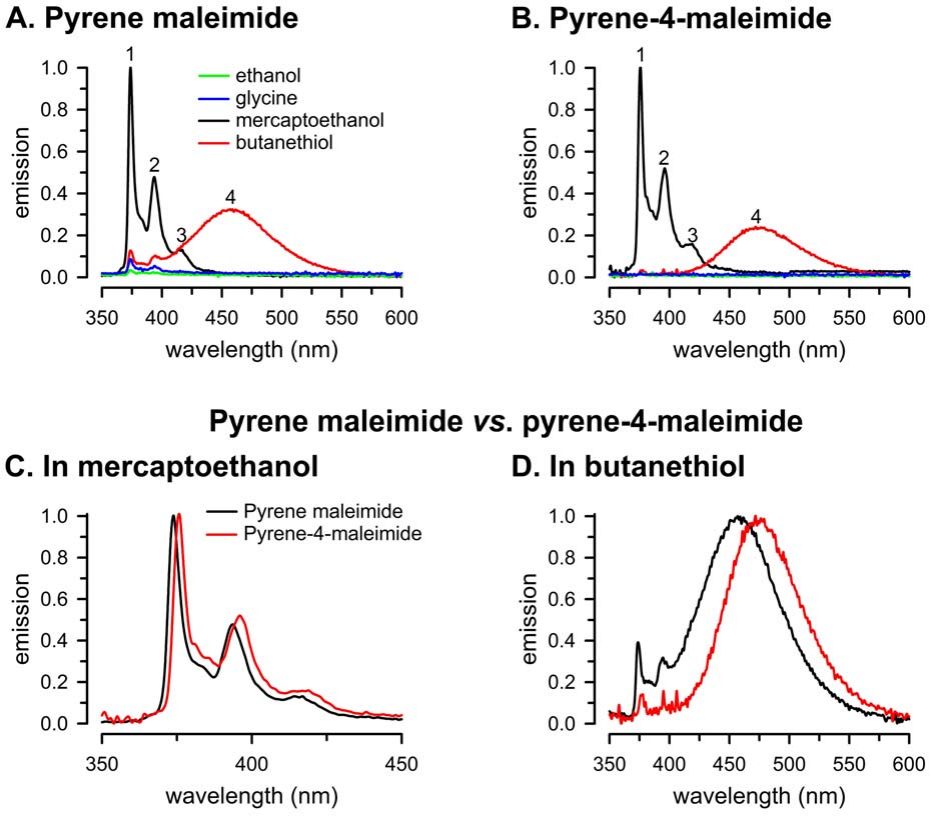
Monomer and excimer emission in the presence of organic compounds. A. Pyrene maleimide. B. Pyrene-4-maleimide. C. Pyrene maleimide *vs.* pyrene-4-maleimide in mercaptoethanol. D. Pyrene maleimide *vs.* pyrene-4-maleimide in butanethiol. The concentrations of the fluorescent probes and organic compounds were 3 µM and 1 mM, respectively. The labels in panels A and C also apply to panels B and D, respectively. Peaks 1–4 are indicated (see text). Data in panels A–C were normalized to peak 1 intensity in mercaptoethanol. Data in panel D were normalized to peak 4 intensity in butanethiol.

Fig. 2 compares the effects on pyrene maleimide and pyrene-4-maleimide fluorescence emission of organic compounds previously tested on the former [15]. Buffer alone (not shown) or addition of 1 mM ethanol or glycine elicited little emission due to a very low fluorescence quantum yield (Fig. 2A and 2B). Addition of 1 mM mercaptoethanol or butanethiol to pyrene maleimide in buffer resulted in a significant increase in emission. However, the effects of these organic compounds on the emission spectra were very different. With mercaptoethanol, the emission is highly structured, with three major peaks of decreasing intensity at the longer wavelengths (labeled 1–3 in Fig. 2A). With butanethiol, the emission is largely unstructured and the main peak (labeled 4 in Fig. 2A) is red-shifted from the third peak with mercaptoethanol by ∼40 nm (Fig. 2A). These results are similar to those previously described [15], and the “mercaptoethanol” and “butanethiol” peaks have been ascribed to emission from monomers and excimers, respectively [1]. The differences between the monomer and excimer emission spectra occur because the excimer emission originates from a lower energy state than the excited monomer.

The emission spectra of pyrene-4-maleimide after addition of ethanol, glycine, mercaptoethanol and butanethiol (Fig. 2B), were generally similar to those of pyrene maleimide. However, there were significant differences. Compared to pyrene maleimide, with mercaptoethanol there is a small but significant red shift of the emission peaks (Δ = 2.3±0.3 nm, P<0.05, Fig. 2C). More noticeable, with butanethiol the excimer peak is ∼20 nm red-shifted compared to the pyrene maleimide peak (Δ = 23±1 nm, P<0.05, Fig. 2D). The intensities of the monomer structured peaks in butanethiol (peaks 1 and 2, the major peaks with mercaptoethanol) were significantly reduced compared to the values of pyrene maleimide. In butanethiol, the relative intensity of pyrene maleimide peaks 1 and 2 (normalized to peak 1 in mercaptoethanol) were 21±6 and 12±3%, respectively. The corresponding pyrene-4-maleimide peaks displayed smaller intensities of 4±1 and 2±0.2, respectively (P<0.05 compared to the corresponding pyrene maleimide peaks, Fig. 2D). The intensities of the excimer emission peaks (also normalized to peak 1 in mercaptoethanol) were similar, at 22±6% (pyrene maleimide) and 18±5% (pyrene-4-maleimide). The red shift of the excimer peak and the decreased intensity of the remaining “monomer” peaks are advantages of pyrene-4-maleimide over pyrene maleimide because they make detection of the excimer emission easier and more sensitive. The integrated emission of pyrene-4-maleimide in mercaptoethanol was slightly less (Δ = −19±7%, P<0.05) than that of pyrene maleimide, but there were no significant differences between the integrated emission between mercaptoethanol and butanethiol (not shown).

Since the emission intensities of pyrene maleimide and pyrene-4-maleimide were very similar (see above), no differences in absorption and quantum yield of the reacted compounds were expected. However, we found that the equivalent emission of the two compounds is the result of the combination of a decreased absorption and increased fluorescence quantum yield of pyrene-4-maleimide *vs*. pyrene maleimide. The pyrene maleimide and pyrene-4-maleimide extinction coefficients (ε) measured in standard buffer with 1 mM mercaptoethanol were 28,564±480 and 7,858±474 M^−1^ cm^−1^, respectively (P<0.05), whereas the fluorescence quantum yield values (Φ_f_), were 0.040±0.002 and 0.131±0.006, respectively (P<0.05). We do not know the reason for these changes, but preliminary data (not shown) with other compounds where the pyrene and maleimide are separated by other linkers produce similar results.

**Figure 3:**
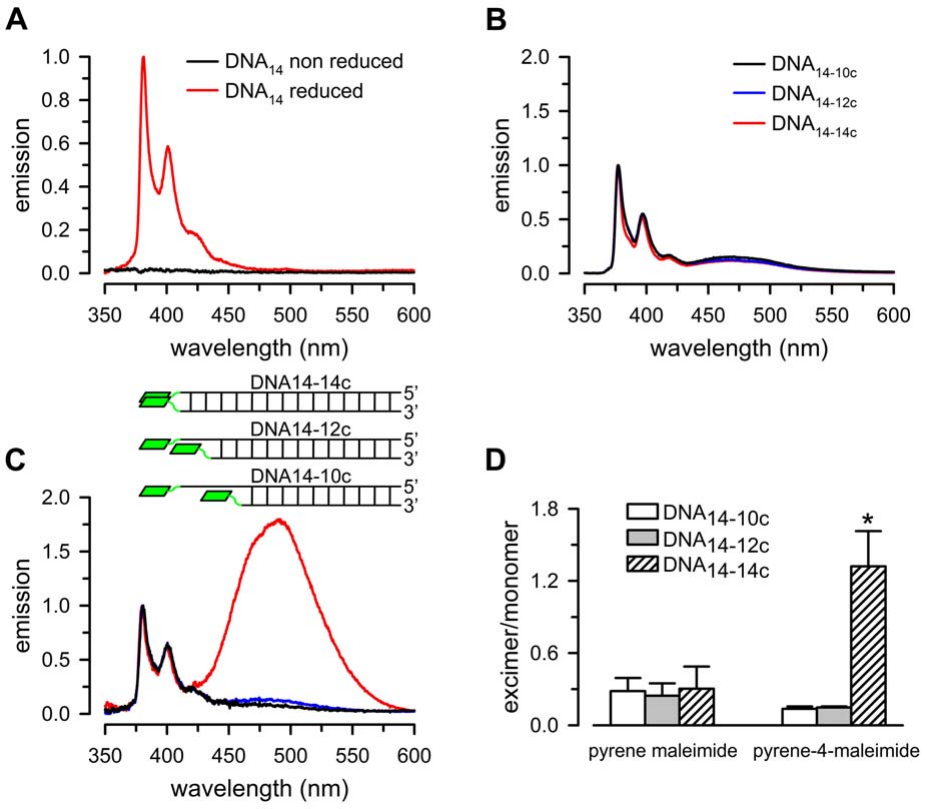
Emission of pyrene compounds reacted with thiol-modified DNA. A. Emission spectra of single-stranded DNA containing a 5′ end thiol group (DNA_14_) reacted with pyrene-4-maleimide. Data were normalized to peak 1 intensity from reduced DNA_14_. [DNA_14_] was 0.5 µM, and [pyrene-4-maleimide] was 2 µM. B. Emission spectra of double-stranded DNA reacted with pyrene maleimide. DNA_14-14c_: DNA_14_ annealed to fully complementary DNA with a 3′ end thiol group (DNA_14c_). DNA_14-12c_: DNA_14_ annealed to a 3′ end two-base shorter complementary oligonucleotide with a 3′ end thiol group. DNA_14-10c_: DNA_14_ annealed to a 3′ end four-base shorter complementary oligonucleotide with a 3′ end thiol group. C. Emission spectra of double-stranded DNA reacted with pyrene-4-maleimide. Insert: schematic representation of the experimental system showing the double-stranded DNAs labeled with pyrene-4-maleimide. The pyrenes are represented by green rhomboids. Data in panels B and C were normalized to peak 1 intensity. The labels in panel B also apply to panel C. D. Excimer/monomer emission ratio. The values were calculated as: excimer/monomer = I_peak 4_/I_peak 1_, where I is the highest intensity of the peak, and peak 1 and peak 4 correspond to the excimer and monomer emission peaks. Averages ± SEM from experiments such as those shown in panels B and C (n = 3 for pyrene maleimide, and n = 4 for pyrene-4-maleimide). The asterisk denotes P<0.05 for the pyrene-4-maleimide DNA_14-14c_ adduct *vs.* each of the other adducts presented in panel D. The concentrations of the fluorescent probes and double-stranded DNA were 4 µM and 0.5 µM, respectively.

The results presented above showed a number of potential advantages of pyrene-4-maleimide *vs*. pyrene maleimide for structural studies of macromolecules. To test the usefulness of pyrene-4-maleimide on an experimental system, we used thiol-modified double-stranded DNAs where one DNA strand was 14 bases long and had a thiol group at the 5′ end (DNA_14_, see Materials and Methods), whereas the complementary strand was either 14 (DNA_14c_), 12 (DNA_12c_) or 10 (DNA_10c_) bases long, and had a thiol group on the 3′ end. Fig. 3A shows that the increase in “monomer” emission that results from the reaction with reduced single-stranded DNA (DNA_14_) is due to specific reaction with the thiol group. Emissions with buffer alone or buffer plus non-reduced DNA were negligible and indistinguishable from each other. Fig. 3C shows that pyrene-4-maleimide-reacted DNA_14-14c_ displays significant excimer emission, which is not the case for the probe reacted with DNA_14-12c_ (Fig. 3C), or the pyrene maleimide-reacted DNA_14-14c_, DNA_14-12c_ or DNA_14-10c_ (Fig. 3B). The results are summarized in Fig. 3D, which displays the data as the ratio of the excimer/monomer peak intensities. The results in Fig. 3 show that pyrene-4-maleimide can sample a longer inter-thiol distance than pyrene maleimide. This could result from the length of the linker, which allows for increased excimer formation at longer inter-thiol distances, and/or its flexibility, which will facilitate pyrene stacking in the correct orientation for excimer emission.

**Figure 4:**
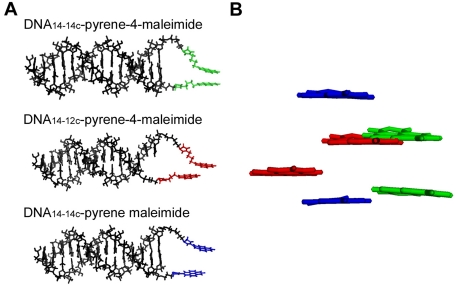
Double-stranded DNA models with pyrene maleimides attached *via* thiol linkers. A. Stick representation view along the DNA long axis. Pyrene-4-maleimide attached to DNA_14-14c_ and DNA_14-12c_ is shown in green and red, respectively. Pyrene maleimide attached to DNA_14-14c_ is shown in blue. The 5′ thiol was present in the 14-bp long strand in all cases. The 3′ thiol was present in the 14-bp or 12-bp long complementary strands. B. Stick representation view perpendicular to that in panel A. Only the pyrenes are shown for clarity. Color coding as in panel A. See Materials and Methods for details.

Fig. 4 displays results from simple models, where the double-stranded DNAs were fixed, and the thiol modifiers and fluorescent probes were energy-minimized. Fig. 4A shows a view along the long DNA axis, whereas the view in Fig. 4B is perpendicular to that in Fig. 4A, and displays only the pyrenes (color-coded as in Fig. 4A). The modeling results show that the pyrenes are in a conformation that can allow excimer formation (on top of each other and ∼5 Å apart) only in the pyrene-4-maleimide DNA_14-14c_ adduct (pyrene-4-maleimide in green). The pyrene-4-maleimide DNA_14-12c_ adduct (pyrene-4-maleimide in red) shows that the increased separation resulting from the shorter 3′-modified DNA strand keeps the pyrenes from stacking on top of each other and ∼10 A apart. In the case of the pyrene maleimide DNA_14-14c_ adduct (pyrene maleimide in blue), the pyrenes are on top of each other, but ∼11 Å apart. They cannot approach each other sufficiently because the fluorescent probe is shorter and more rigid than pyrene-4-maleimide. The modeling data are consistent with the experimental results in Fig. 3, where only the pyrene-4-maleimide DNA_14-14c_ adduct displays significant excimer emission. Compared to the emission in butanethiol, reaction of pyrene-4-maleimide with double-stranded DNA_14-14c_ showed the same peaks, but with a red shift, smaller for the monomer (Δ = 2.7±0.1 nm) than for the excimer peak. The excimer emission of pyrene-4-maleimide bound to DNA_14-14c_ (Fig. 3C) peaked at 489±1 nm (compared to 470±0.2 nm for the excimer peak in butanethiol, P<0.05, Fig. 2D). This spectral shift is a favorable effect that makes pyrene-4-maleimide easier to detect.

Reaction of macromolecule thiols with maleimides depends on the accessibility of the reactive groups and reactivity in the medium in which the thiols are located. The data presented so far show that pyrene maleimide and pyrene-4-maleimide react well with thiols in water. We also tested the reaction of these two maleimide derivatives (5 µM) with dithiothreitol (5 µM) in dimethylsulfoxide. The reaction, followed by the increase in fluorescence at 370 nm (340 nm excitation), was complete after mixing (<5 s). Even though accessibility of the maleimides will always be a potential problem, our data show that pyrene-4-maleimide reacts well with thiols in a solvent with lower dielectric constant than water.

**Figure 5:**
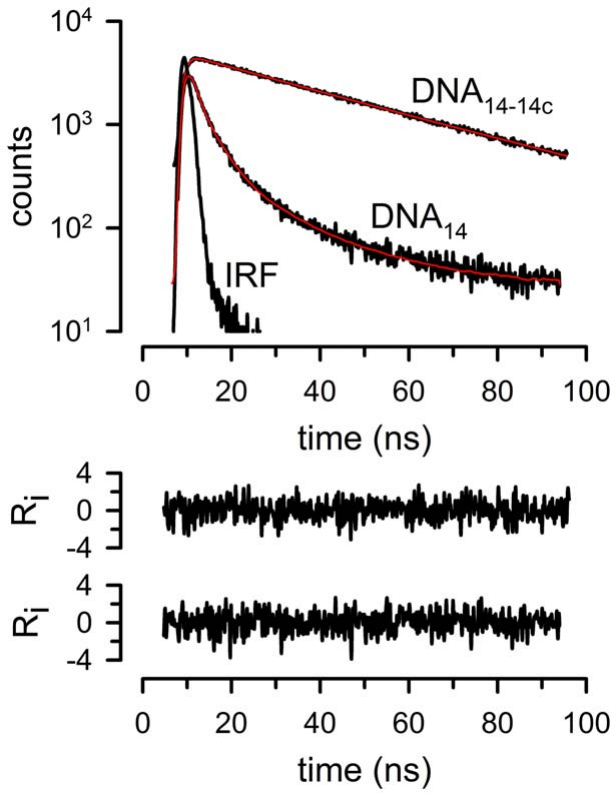
Long lifetime of pyrene-4-maleimide excimer emission. DNA_14_: reduced single-stranded DNA_14_ adduct. DNA_14-14c_: double-stranded DNA_14-14c_ adduct. IRF: instrument response function. The red lines are fits of the data to multi-exponential functions, with the two weighted residuals (R_i_) *vs.* time plots corresponding to the double-stranded (top) and single-stranded (bottom) data fits.

Fig. 5 shows typical emission lifetimes of single-stranded (DNA_14_) and double-stranded DNA (DNA_14-14c_) reacted with pyrene-4-maleimide. The lifetime of the DNA_14_ monomer emission was significantly shorter than that of the excimer from the DNA_14-14c_. Exponential fits of the DNA_14_-pyrene-4-maleimide intensity decays (labeled DNA_14_) showed the presence of three lifetimes of 0.8±0.1 (13±5%), 2.7±0.5 (51±1%) and 10.7±1.3 ns (37±6%). The DNA_14-14c_-pyrene-4-maleimide decay (labeled DNA_14-14c_) consisted of a main component (93±3%) of longer lifetime (33.6±1.0 ns, P<0.05, n = 4, compared to the longest lifetime in DNA_14_), and a small faster component (2.3±0.04 ns, 7±3%). Long pyrene excimer lifetimes have been previously reported [2], [5], [15]. The clear difference between monomer and excimer emission decays of pyrene-4-maleimide and the relatively long lifetime of the excimer emission allows for easy identification of excimer emission independent of the sample intensity.

In summary, the results show a number of advantages of pyrene-4-maleimide over pyrene maleimide: 1) It can detect proximity between thiols that are beyond the reach of pyrene maleimide. This property makes pyrene-4-maleimide a better probe for most applications, and constitutes an excellent complement of pyrene maleimide to detect conformational changes in proteins. For example, if pyrene maleimide excimers are lost as a result of a conformational change, the continuous presence of pyrene-4-maleimide excimers will suggest that the increased distance between the thiols changed by <12 Å (twice the average distance of the pyrene-4-maleimide linker). 2) The flexibility of the linker between the pyrene and the maleimide can increase the efficiency of excimer emission. It can allow for better alignment of the pyrenes to form excimers, and facilitate reorientation during excitation to reduce quenching. 3) The increased red shift of the excimer peak makes detection of excimer fluorescence simpler and more sensitive because it moves the excimer peak away from the shorter wavelength “monomer” peaks. 4) The long excited-state lifetime of the pyrene-4-maleimide excimers *vs.* monomers emission, make the use of pyrene-4-maleimide lifetime excimer detection possible and relatively simple. Intensity-independent excimer detection is very useful in cases where background complicates measurements of intensities. From the properties described above, we suggest that pyrene-4-maleimide is an excellent probe to assess proximities between cysteine in proteins and thiols in other macromolecules, and to follow conformational changes.

## Materials and Methods

### Reagents

All reagents used were of the highest available quality. Pyrene maleimide (Invitrogen, Carlsbad, CA) and pyrene-4-maleimide stock solutions (25 mM) were prepared in dimetylsulfoxide. Mercaptoethanol and butanethiol were purchased from Sigma-Aldrich (St. Louis, MO). When used, the concentration of these organic compounds, as well as that of ethanol and glycine, was 1 mM in our “standard” buffer: 200 mM KCl and 20 mM Tris/HCl, pH 7.5.

### Modified DNA oligonucleotides

We used a sense oligonucleotide of the following sequence: 5′-CATCGTAGAGGCAG-3′, with a 5′ thiol modifier C6 S-S (DNA_14_). The fully complementary oligonucleotide sequence 5′-CTGCCTCTACGATG-3′ had a thiol group at the 3′ end (3′ thiol modifier C3 S-S, DNA_14c_). We also used two additional DNA_14_ complementary oligonucleotides that lacked the first two (DNA_12c_) and four (DNA_10c_) bases at the 3′ end. Assuming an ideal double-stranded helix, the DNA_12c_ and DNA_10c_ strands are 6.6 and 13.2 Å shorter, respectively, than the DNA_14c_ strand. The oligonucleotides purified by HPLC were purchased from Integrated DNA Technologies (Coralville, IA), and were dissolved in our standard buffer. After reduction of the thiols with tris(2-carboxyethyl)phosphine (TCEP, 1 or 5 mM for 1 h at room temperature), TCEP was removed by gel filtration on Illustra G-25 mini-columns (GE Healthcare, Piscataway, NJ). The reduced single-stranded DNAs were quantified by absorbance, mixed at a 1∶1 molar ratio, and annealed by heating to 94°C for 20 min, with a subsequent slow cool down to room temperature. Hybridization was checked on 5% agarose gels. One advantage of the pyrene maleimides is that they display very low fluorescence in water-based solvents, but experience a dramatic increase in fluorescence quantum yield upon reaction with thiols. In most experiments, pyrene maleimide and pyrene-4-maleimide were added to the DNA solutions and measurements were performed after the increase in fluorescence reached stable values (<10 min). The results were similar to those in experiments where the unreacted probes where removed by gel filtration after labeling (not shown). The fluorescent probes were used at an 8-to-1 molar ratio; generally 4 µM fluorescent probe and 0.5 µM double-stranded DNA.

### Synthesis of pyrene-4-maleimide

Synthesis was performed as follows: A 25 mL round bottom flask was charged with triphenylphosphine (Ph_3_P) (86.8 mg, 0.331 mmol) and tetrahydrofuran (2.24 mL), and the resulting clear solution was cooled to −78°C in a dry ice-acetone bath. Diisopropyl azodicarboxylate (DIAD) (66.9 mg, 0.331 mmol) was added to this mixture over 2–3 min, and the resulting yellow mixture was stirred for 5 min. Then, 1-pyrenebutanol (100 mg, 0.364 mmol) was added over 1 min and stirred for 5 min. Maleimide (32.1 mg, 0.331 mmol) and neopentyl alcohol (16 mg, 0.182 mmol) were then added to the reaction mixture, and the resulting suspension was maintained at −78°C for 5 min, a time during which most maleimide was dissolved. The reaction mixture was removed from the cooling bath and stirred overnight at room temperature. The resulting clear solution was concentrated under vacuum, and the residue was purified by silica-gel column chromatography (hexane:ethyl acetate, 12∶1), followed by preparative TLC to obtain pure *N*-pyrenylbutyl maleimide (12.3 mg, 0.035 mmol).

*N*-pyrenylbutyl maleimide (pyrene-4-maleimide) yield and properties were as follows: yield 10.6%. Mp 97–98°C. ^1^H NMR (500 MHz, CDCl_3_): δ 1.74–1.81 (m, 2H), 1.81–1.89 (m, 2H), 3.37 (t, 2H), 3.60 (t, 2H), 6.65 (s, 2H), 7.85 (d, 1H), 7.96–8.20 (m, 7H), 8.25 (d, 1H). ^13^C NMR (125 MHz, CDCl_3_): δ 28.52, 28.79, 32.90, 37.67, 123.31, 124.70, 124.81, 124.86, 125.00, 125.07, 125.80, 126.62, 127.28, 127.29, 127.49, 128.58, 129.86, 130.88, 131.41, 134.02, 136.18, 170.85. HRMS *m/z* calculated for C_24_H_19_NO_2_ (M)^+^ 353.1416, found 353.1410.

### Spectrophotometry and steady-state fluorescence measurements

Excitation and emission fluorescence spectra were measured on a Hitachi F-7000 (Tokyo, Japan) or Photon Technology International QM3SS (London, Ontario) spectrofluorometers. Generally, excitation and emission slits were 1.5 nm. Absorbance was measured on a Shimadzu spectrophotometer UV160 (Kyoto, Japan).

### Quantum yield measurements

Fluorescence quantum yields (Φ_f_) were determined relative to anthracene in methanol. This compound was chosen because it absorbs well at the pyrene maleimide and pyrene-4-maleimide excitation wavelength (340 nm) and emits in the same wavelength range as the pyrene fluorophores [23]. Generally, we prepared four different concentrations of anthracene in methanol, and pyrene maleimide and pyrene-4-maleimide (in standard buffer with 1 mM mercaptoethanol), keeping absorbance at 340 nm lower than 0.1. Under these conditions, there was a linear relationship between absorbance and total integrated emission, and therefore no corrections for inner filter effects were performed. The relative quantum yields were taken as the slopes of the linear absorbance *vs*. emission integrals, and converted to fluorescence quantum yields using an anthracene Φ_f_ of 0.3 [23].

### Fluorescence lifetime measurements

Lifetimes were measured in the time-domain with an ISS ChronosBH lifetime spectrometer (Champaign, IL). Excitation was from a 335 nm pulsed LED (5 MHz) and single-photon counting detection was through a 440-nm short-pass filter (FF01-440/SP, Semrock, Lake Forest, IL) for monomer emission, or a 450-nm long-pass filter (450LP, Newport, Irvine, CA) for excimer emission, with the excitation polarizer at 0° and the emission polarizer at the magic angle (54.7°). Anthracene in methanol, 9-anthracenecarbonitrile (9-cyanoanthracene) in methanol, and 10-(3-sulfopropyl)acridinium betaine in water were used as instrument test compounds because they give essentially single-exponential decays with lifetimes that cover the range of those measured with pyrene-4-maleimide [23]. The instrument response function was measured with Ludox in water as a scatterer, and multi-exponential curve fitting was done with the Vinci Analysis software.

### Modeling of modified DNAs

DNA models were constructed using 3D-DART [24], and the pyrene maleimide and pyrene-4-maleimide adducts were generated in Avogadro. Each of the resulting hybrid models was energy-minimized in Avogadro using the Ghemical force field, with the atoms in the DNA fixed to avoid distortion of the bases. Figure 4 was rendered with PyMol.
